# The effect of a tailored message package for reducing antibiotic use among respiratory tract infection patients in rural Anhui, China: a cluster randomized controlled trial protocol

**DOI:** 10.1186/s13063-023-07664-8

**Published:** 2023-10-04

**Authors:** Rong Liu, Qun Xue, Xiaoqin Guan, Guocheng Li, Tingting Zhang, Debin Wang, Linhai Zhao, Xingrong Shen

**Affiliations:** 1 School of Health Services Management, Anhui Medical University, Hefei, Anhui China; 2 Bristol Medical School, Population Health Sciences, University of Bristol, Bristol, BS8 2PN UK; 3 Center for Appropriate Technology Research in Health Services and Management, Anhui Medical University, Hefei, Anhui China

**Keywords:** Antibiotics, Messages, Patient education, China, RCT

## Abstract

**Background:**

Antibiotics are over-used for patients with respiratory tract infections (RTIs) in primary care, especially in the rural areas of China.

**Methods:**

A cluster randomized controlled trial (RCT) will be carried out to estimate the effectiveness of a tailored message package for educating patients to reduce antibiotic use for symptomatic respiratory tract infections (RTIs). In the intervention group, patients will receive 12 short messages in 12 consecutive days. The whole process of the message design, modification, translation (of substitution variables), and sending will be facilitated by a user-friendly mini-computer program. The primary measure for assessment is the reduction in number of days in which antibiotics are used by patients with symptomatic RTIs. The secondary measures include (1) patients’ knowledge about and attitude toward antibiotics; (2) patients’ quality of life (EQ-5D-5L) and symptom severity and duration; (3) times of re-visits to clinics and antibiotics re-prescription for the same RTI episode; and (4) times of re-occurrence of RTIs and related health service seeking and antibiotics consumption.

**Discussion:**

This study will determine the efficacy of a 12-message intervention to educate patients to reduce excessive antibiotic use in rural China.

**Trial registration:**

ISRCTN29801086. Registered on 23 September 2022.

**Supplementary Information:**

The online version contains supplementary material available at 10.1186/s13063-023-07664-8.

## Background

Antibiotic resistance (ABR) has become an urgent public health problem worldwide [1]. China is among the largest countries of antibiotics consumption and excessive use in primary care is most prominent. The proportion of antibiotic use in patients with symptomatic RTIs is estimated to be over 80% [2–9]. Globally, about 700,000 people died of antibiotic resistance in 2014 and this figure will reach 10 million per year by 2050. If effective prevention and control measures are not taken, drug-resistant infections will cause economic losses of about 100 trillion US dollars [10].

A variety of measures have been taken in China to improve antibiotic use [11–15]. These include the introduction of antimicrobial drug monitoring networks, practice guidelines, prescription formularies, restrictions on antibiotic prescription authority for doctors at different levels, education of doctors and patients, and others. Most of these approaches have showed some effects [16]. However, contemporary efforts have been focused primarily on doctors, with far less attention being put on patients [17, 18]. Antibiotic prescription reflects the joint interaction between the doctor and patient. Although the doctor dominates most of the consultation encounters, the patient also has important direct and/or indirect influences on the prescription decisions. Doctors often attribute excessive antibiotic use to “patient pressure and expectations” or “patient demand” [19]. They feel that their patients may be more satisfied if they were prescribed some medicine even if it is unnecessary. However, over 70% of patients reported that they would comply with their physician’s decision of not giving any prescription [20].

According to our previous studies and others, patients in China have various beliefs and habits that lead to excessive use antibiotics. They lack a general understanding of uses, especially resistance and other side effects, of antibiotics [21]. Some of them believe that antibiotics help in treating almost all diseases. Although China policy requests that (since 2020) all antimicrobials must be dispensed with a prescription in all retail pharmacies [22, 23], it is not fully enacted in rural areas [24, 25]. As a result, patients can easily get antibiotics from retail pharmacies without prescriptions. In addition, most consultations in China are very short and doctors seldom have time or desire to discuss antibiotics with their patients, while patients on the other hand seldom have the opportunity to explicitly express their expectations about antibiotics. These all point to a clear need for patient education. This study is an innovative attempt in this regard.

## Methods/design

The trial protocol was designed according to the SPIRIT reporting guidelines [26].

### Aim and objectives

#### Aim

This study aims to establish and validate a tailored message package (TMP) for reducing antibiotic use among patients clinically diagnosed with RTIs.

#### Objectives

1. To determine the effectiveness of the TMP in terms of reduced duration of antibiotic usage among patients with RTIs compared with those in the usual care (UC) arm.
2. To compare knowledge and attitudes about antibiotics and use or reuse of health care for this and recurrent RTI episodes among patients between the TMP and UC groups.
3. To assess the difference in quality of life between the TMP and the usual care arm

### Study settings

**Fig. 1:**
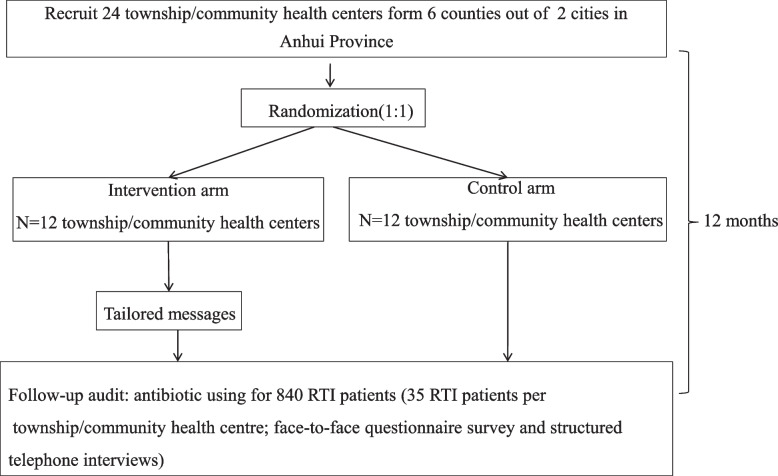
Trial flow chart

The RCT will be implemented in Anhui, an inland province in China. The study sites are township or community health centers in the province which are selected through the following steps: (1) select 2 cities from the province using convenience sampling; (2) randomly select 3 counties from each of the cites selected; (3) randomly select 4 nonadjacent township or community health centers from each of the selected counties (Fig. 1); and (4) randomly select equal number of township or community health centers from the none selected centers within the same county to substitute those that were selected via previous step yet declined to participate.

### Trial design

The study adopts a cluster randomized controlled trial (RCT) design, involving two arms of equal townships or community health centers, i.e., the TMP arm or the UC arm.

### Allocation to study arms

The recruited township or community health centers will be randomly allocated to TMP and UC arms at the same time after the completion of baseline data collection. The allocation process will be performed by an external statistician via 4 steps. Step 1, the project facilitator sends, via email, the allocation requirements alongside the name-list of the 6 selected counties and the 4 health centers recruited under each of the counties to the external statistician. Step 2, the statistician performs the random assignment of the health centers into equal arms of TMP and UC using the “random sample of cases” function in IBM SPSS on a county-by-county base so that the 4 health centers recruited from each of the site counties is randomly assigned as 2 to the TMP arm and the other 2 to the UC arm. Step 3, the statistician emails back the resultant allocation to the project facilitator. Step 4, the facilitator communicates the allocation to the participating physicians and arranges the planned training for those in the TMP arm.

### Blinding

Given the nature of the intervention, doctors, telephone interviewers, and site investigators will not be blinded to the assigned condition since TMP is mentioned during the patient-doctor encounters and in the interviews. However, the statisticians responsible for the analysis will be blinded to the allocation until the quantitative analysis has fully completed. Unblinding is not relevant to this trial.

### Interventions

#### Usual care

The intervention will be tested against usual care. In other words, the control group will maintain usual care, while the intervention group patients will receive, in addition to usual care, 12 short messages in 12 consecutive days after the consultation. Here, usual care refers to the existing procedures (mainly, history inquiries, physical checkup, lab tests, clinical diagnosis, and prescribing treatment) being routinely practiced in the consultation and management of patients with RTIs. Using UC as the control condition is advantageous in at least a couple of senses: it involves almost no further changes in the control group and is thus most easy to implement the trial; it allows to assess the added value of the TMP upon the existing care and should thus meet with the least barriers in disseminating the trial results.

#### Text messages package

Eligible patients in the intervention condition will receive, via mobile phones, a package of 12 short (less than 120 Chinese characters) messages in 12 consecutive days (once a day). Detailed content of the messages is given in Table 1. Each of these messages was based on the main drivers of excessive antibiotics as identified from our previous studies [27] and has specific aims. For example, the message, to be sent out on day 1, aims to build trust and assure the patient not to worry about common RTI symptoms and most of which will start to mitigate within a few days and disappear within about 1 week. Similarly, the message to be sent on day 2 aims mainly to provide alternative ways for coping with RTI symptoms so as to divert his/her reliance on medications. While the message on day 3 tells the patient to postpone or avoid uptake of the antibiotics prescribed by his/her doctor.

**Table 1:** Messages to be sent out to patients with symptomatic respiratory infections

**No.**	**Content of message**
Day 1	Dear [PatientName], many thanks for your trust and visit to our clinic for a [DiseaseDiagnosis]. What you were experiencing were mainly [PatientSymptom]. These are very common symptoms. They generally reach a peak within 1-3 days and then begin to relieve gradually. You needn’t see any doctor again unless you do not get better within [NormalDuration]. From [DoctorName] with [ClinicName].
Day 2	Dear [PatientName], the best way to deal with most respiratory tract problems such as [DiseaseDiagnosis] is to improve your own body immunity and what you need to do are only the following. First, eat more protein-rich and digestible foods, such as fish or egg soup. Second, eat more fresh vegetables, fruits, garlic and ginger. Third, have more rest and drink more warm water. From [DoctorName] with [ClinicName].
Day 3	Dear [PatientName], most of the respiratory tract health problems such as [DiseaseDiagnosis] do not need antibiotics, such as amoxicillin, cephalosporin, and others. The medications your doctor has prescribed for you are to be used only when there are clear indications. In other words, you need only take fever pills when you have high fever; you need only take antibiotics when your symptoms do not recover after [NormalDuration]. From [DoctorName] with [ClinicName].
Day 4	Dear [PatientName], [DiseaseDiagnosis] is easy to transmit to family members and friends. Please avoid contact with them, especially children and the elderly in the local community for 1-2 weeks. Please practice ventilating your living rooms, wearing masks when you go out or meet with people, avoiding eating out and going to crowded places. From [DoctorName] with [ClinicName].
Day 5	Dear [PatientName], most respiratory tract infections such as [DiseaseDiagnosis] are caused by virus. Generally, they are self-limiting without oral medicines or injections. Antibiotics do not work for viral infections, which can neither ease the severity of symptoms nor shorten the duration. Most importantly, antibiotics can cause antibiotic resistance and various other side effects. From [DoctorName] with [ClinicName].
Day 6	Dear [PatientName], taking antibiotics (such as amoxicillin and cephalosporin) will weaken your own body immunity, and will kill the good bacteria in your gut as well, or upset the balance of/in your stomach. The more antibiotics you take, the higher chance that you suffer from an antibiotic-resistant-infections. This infection is not only serious, but also difficult to treat and are easy to transmit to family members. From [DoctorName] with [ClinicName].
Day 7	Dear [PatientName], when you feel unwell with respiratory tract, you should not go to retail pharmacies to buy antibiotic. China National Medicine Administration Bureau has banned pharmacies to sell antibiotics to residents without a doctor's prescription. China National Health Commission advises not to have a drip or take antibiotics for coughs, cold, sore throat or earache. From [DoctorName] with [ClinicName].
Day 8	Dear [PatientName], are you feeling any better from your [DiseaseDiagnosis]? If yes, you seem to be recovering as expected; If not, you should better see your doctor again. As antibiotics do almost no good for respiratory tract infections but have many side effects, we recommend you clearly tell your doctor that you don't want antibiotics whenever you consult a doctor in the future; or, if you are prescribed with antibiotics, ask your doctor whether the antibiotics can be avoided. From [DoctorName] with [ClinicName].
Day 9	Dear [PatientName], your current illness suggests that every on including you can get respiratory tract infections. Therefore, you need to strengthen your personal protection in the future. This include: eat more fresh vegetables and fruits; exercise more; wash hands more frequently; avoid crowded places when many people around you are having respiratory symptoms and wear a mask when you go out. From [DoctorName] with [ClinicName].
Day 10	Dear [PatientName], a certain proportion of respiratory tract infections are influenza. Influenza is highly contagious and easy to increase the extra risk of common health conditions like chronic respiratory tract diseases and cardiovascular and cerebrovascular diseases. Therefore, the State encourages residents to get influenza vaccination, especially for the elderly over 60 years old, patients with chronic diseases, children aged 6 months to 5 years and caregivers for infants under 6 months. From [DoctorName] with [ClinicName].
Day 11	Dear [PatientName], after recovery from your current illness, if you have respiratory symptoms next time in the future, please do not hurry seeing a doctor but wait and watch its progression, do not go to retail medicine shop to buy antibiotics without a prescription, and do not take leftover antibiotics. Please practice rest at home, drink more water and eat more protein-rich and digestible food and more fresh vegetables. Most respiratory infections are self-limiting and the symptoms start to relief within a few days. From [DoctorName] with [ClinicName].
Day 12	Dear [PatientName], this is the last message we plan to send you. We hope you will do as what the messages have suggested and avoid antibiotics use whenever possible. Antibiotics have no effect on most respiratory infections - they cannot relief the severity of symptoms or shorten the disease duration. On the contrary, antibiotics are easy to cause drug resistance and various other side effects. Thank you for accepting and reading all the messages and all the best wishes for you. From [DoctorName] with [ClinicName].

#### Message development

The above messages originated from a multiple-stage development. First, a literature review was performed to identify published knowledge, attitude, and behavior barriers to rational use of antibiotics among RTI patients and measures used to tackle these barriers. Second, tentative lists of the barriers and corresponding messages helpful in overcoming the barriers were generated based on the literature review and results from our previous research findings. Third, two rounds of consensus meetings were organized to select, refine, and form a preliminary message package from the lists of barriers and counter messages. Fourth, “think-aloud” interviews with the presenting RTI patients and their doctors at 6 selected township and community health centers were carried out to test the comprehensibility and acceptability of the preliminary message package. Fifth, a final 12-item message package was derived based on these “think-aloud” interviews.

#### Message tailoring

The study uses a novel computerized method in tailoring the messages to the need and context of individual patients. More specifically, each of the messages is designed as a template inserted with substitution variables to be replaced with relevant values and/or text according to the actual conditions and contexts of the patient under concern. Taking the example of the message to be sent on day 1 (Table 1), it contains seven variables specified as “[PatientName],” “DiseaseDiagnosis,” etc. When sent to a patient, for example, named “Li Si,” who has consulted doctor Zhang San at “SanXiaoKou Community Health Center” for sneezing, stuffy nose, and sore throat and the doctor diagnosed the illness as “common cold.” The message is changed to: “Dear Li Si, many thanks for your trust and visit to our clinic for a common cold. What you were experiencing were mainly sneezing, stuffy nose and sore throat. These are very common symptoms. They generally reach a peak within 1-3 days and then begin to relieve gradually. You needn’t see any doctor again unless you do not get better within 14 days. From Dr Zhang San with SanXiaoKou Community Health Centre.”

Most of the substitution variables are determined by built-in computerized algorithms based on literature reviews or findings from the research team’s previous investigations and the expert consensus mentioned earlier. Taking the example of the “normal duration,” the online system has a built-in “dictionary” which reads like {“diagnose 1”: duration 1, “diagnose 2”: duration 2, …, “diagnose *n*”: duration *n*}. So, the online system can automatically provide a relevant duration value upon the diagnose entered by the doctor.

#### Message sending

**Fig. 2:**
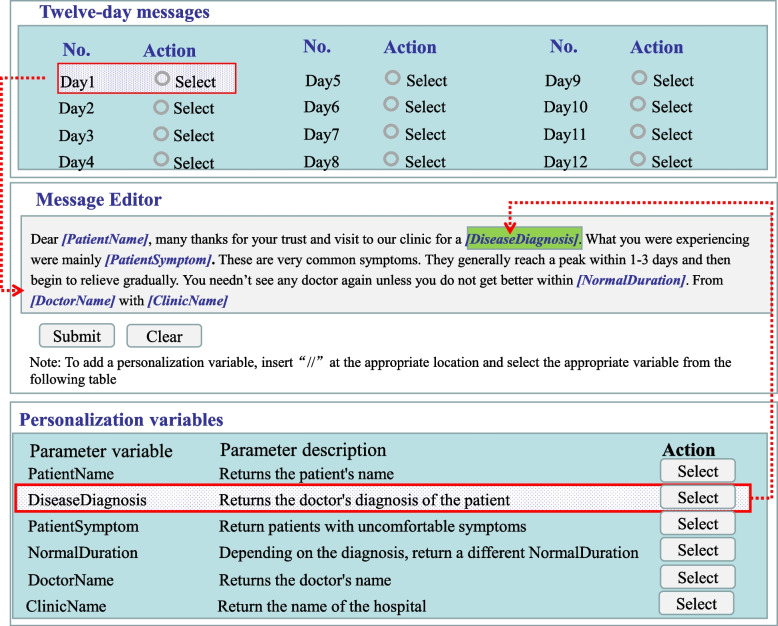
Screen print of the mini computer program for designing and tailoring intervention messages

The whole process of the message selection, modification (substitution of embedded variables), and sending will be by a user-friendly mini computer program. The program automatically extracts data about the patient’s name, symptoms, and diagnosis from the electronic medical record system routinely in use at all the participating clinics and then translates all the substitution variables into personalized texts. So, implementation of the intervention incurs little additional workload for the participating doctors. What the doctors need to do is limited to obtaining informed consent and cell phone numbers from the patients and adding them into the list eligible to receive the TMP; Fig. 2 illustrates how the mini program works. The upper part of the figure provides a name list of all the messages to be sent out to patients. The middle part shows an example message template and the doctor is allowed to modify the template for his/her own patients, while the lower part presents all the substitution variables capable of being automatically translated.

#### Intervention adherence

Pragmatic measures will be used to improve intervention adherence from both doctor and patient perspectives. For doctors, adherence will be enhanced by (1) one session training of doctors in the intervention arm on TMP content and benefits; (2) user-friendly online system which minimizes the doctors’ workload in using the TMP; (3) automatic recording and presentation, via the online system, of individual doctor’s intervention performance (e.g., absolute number and percent of RTI patients who have been sent the TMP) as compared with his/her peers; and (4) award of continued education credits for doctors who meet a preset performance standard (e.g., delivery of TMP to 80% of the RTI patients encounters during the study period). Similarly, measures for improving patient compliance will include the use of tailored and easily comprehensible TMP, delivery of highly relevant messages during a “time-window” when the patients are seeking professional help, and prior advice by the doctors during the initial consultation on the need to read the TMP in the forthcoming days.

### Trial evaluation

#### Outcome measures

The primary outcome is the number of days of antibiotics use, including antibiotics obtained before and after the initial consultation. The secondary outcomes include (1). illness duration and severity, (2) patients’ attitude and understanding of intervention messages, (3) patients’ quality of life, (4) re-visits and antibiotic usage, (5) patients’ knowledge and attitude toward antibiotics, and (6) recurrence of RTIs and related health service and antibiotics use. Definition and calculation of these measures are summarized in Table 2.

**Table 2:** Primary and secondary measures for trial evaluation

Measure	Definition/calculation
**Primary measure**	
-Number of days with antibiotics use	Days of antibiotics use from onset of the RTI to the initial consultation + days of antibiotics use from day 1 to 21 after the initial consultation based on the patient’s responses to question item A5d in Additional file 1, B2 in Additional file 2, and item C2-C4 in Additional file 3. Here, days of antibiotics use is defined as days on which the patient has used any antibiotics.
**Secondary measures**	
-Illness duration	Days from the initial consultation to the day when the patient has reported full recovery based on the patient’s responses to question item A3a in Additional file 1, item B1b in Additional file 2, and item C1b in Additional file 3.
-Illness severity	Ten-point Likert rating of symptom severity based on the patient’s responses to question item A3b in Additional file 1, item B1c in Additional file 2, and item C1c in Additional file 3.
-Times of re-visits	Times of re-visits to the same clinic as the initial consultation + visits to other clinics for the same RTI episode based on the patient’s responses to question items C3a1, C4b12, and C4b22 in Additional file 3.
-Antibiotics re-prescription	Times of antibiotics re-prescription during the patient’s re-visits to the same clinic as the initial consultation and visits to other clinics for the same RTI episode based on the patient’s responses to question items C3a2, C3a3, C3a4, C4a3, C4b15, and C4b25 in Additional file 3.
-Knowledge about and attitude toward antibiotics	Percentage of correct responses to the questions relating to knowledge and attitude about antibiotics, i.e., item D1 in Additional file 4.
-Times of re-occurrence of RTIs	Times of re-occurring RTI episodes 180 days or 365 days following the initial consultation based on the patient’s responses to question item D2a1 in Additional file 4.
-Health service seeking for re-occurred infections	Times of visits to clinics for re-occurring RTI episodes 180 days or 365 days following the initial consultation based on the patient’s responses to question item D2b1-D3c41 in Additional file 4.
-Antibiotics consumption for re-occurred infections	Times of antibiotics prescriptions for re-occurring RTI episodes 180 days or 365 days following the initial consultation based on the patient’s responses to question items D2b3, D2c3, and D3c3 in Additional file 4.
-Quality of life (EQ-5D-5L)	Quality-adjusted life years based on the patient’s responses to the EQ-5D-5L scales in Additional file 5.
-Attitude toward intervention messages	Percentage of ideal responses to the questions relating to attitude toward the intervention messages, i.e., item B3d-B3e in Additional file 2, item C5d-C5e in Additional file 3.
-Understanding of intervention messages	Percentage of ideal responses to the questions relating to the understanding of the intervention messages, i.e., item B3c in Additional file 2, item C5c in Additional file 3.

#### Sample size

The sample size of patients to be recruited for the trial evaluation is calculated on the basis of the primary outcome measure above. Our previous study [18] estimated that the number of days of antibiotic use by symptomatic RTI patients was 4.35 $\pm$ 2.06 for rural township health centers in Anhui province. There is no internationally agreed minimally important difference in the days of patients using antibiotic. However, we anticipate that a reduction of at least 15% in the days of antibiotic use would represent a meaningful change at the population level. So, we assume the following: (1) the intervention group is 20% lower than the control group, i.e., a reduction of about 0.87 days; (2) standard deviation of the days of antibiotics use is 2.06 days; and (3) the alpha value is 0.05. Given these, to detect a possible absolute difference of 0.87 days in the rates with 90% power, we will need 118 RTI patients in each arm. Based on our previous study results the estimated design effect value would be 2.45. By allowing for a 20% attrition rate and for a 20% loss to follow-up rate, therefore we would aim to recruit at least 2.45×118×2×1.2×1.2=833 patients into the study and this translates into about 35(833/24=35) patients per township/community health center.

#### Eligibility criteria

The inclusion criteria for patients are presenting males or females to the site health centers who are (1) 18 years or older and able to give consent to participate in the patient survey and/or follow-up interviews; (2) diagnosed, by doctor, with common RTIs, including acute upper RTI, common cold, acute bronchitis/tracheitis, exacerbation of chronic obstructive pulmonary disease, and influenza-like illness; and (3) able to receive and comprehend messages via either self-reading or assistance from family members. Exclusion criteria are patients who (1) have previously sought treatment for the current illness from the participating health centers and (2) are pregnant individuals.

#### Patient recruitment

In order to evaluate the trial, 35 patients diagnosed with RTIs from each of the site township or community health centers will be recruited using a consecutive strategy. More specifically, each of the health centers in the intervention and control arms will be sent one investigator and from the start date when he/she arrives at the site, all incoming patients with RTIs will be invited to participate after consultation until a preset number of eligible patients have been recruited. The field investigator will conduct the recruitment face-to-face after obtaining informed consent from participants.

#### Data collection

The study data will be collected through one baseline and five follow-up telephone interviews. For any recruited patient, the baseline interview happens immediately after the consultation, while the follow-up interviews are scheduled at 7, 14, 21,180, and 365 days after the baseline. The interviews will be performed by trained data collectors using a structured questionnaire adapted from our previous study [18] (Additional files 1, 2, 3 and 4). The main content of the questionnaire includes patient demographic characteristics (e.g., sex, age, education, residential area, and medical insurance status), illness duration, symptoms, severity rating, diagnosis, antibiotics, and other medicines prescribed. EPI DATA 3.1 will be used to create a data entry interface for all the on-site and telephone follow-up interviews. Table 3 summarizes measure by measure time schedule for data collection.

**Table 3:** SPIRIT schedule of outcome measures

**Category**	**Outcome measure**	**Initial consultation**	**Days after consultation**				
			**7**	**14**	**21**	**180**	**365**
Primary measure	Number of days with antibiotics use	√	√	√	√		
Secondary measures	Illness duration	√	√	√	√		
	Illness severity	√	√	√	√		
	Times of re-visits				√		
	Antibiotics re-prescription				√		
	Knowledge about and attitude toward antibiotics					√	√
	Times of re-occurrence of RTIs					√	√
	Health service seeking for re-occurred infections					√	√
	Antibiotics consumption for re-occurred infections					√	√
	Quality of life (EQ-5D-5L)	√	√	√	√		
	Attitude toward intervention messages		√	√			
	Understanding of intervention messages		√	√			

#### Data quality control

All field and telephone interviewers will be trained on the content of the face-to-face and telephone interviews and principles and tips for performing the interviews before data collection. Embedded logical examinations will be designed into the aforementioned EPI DATA data entry tool to prevent missing fields and out-of-range values. All interviews will be, after informed consent, audio recorded to allow for later self and independent quality checks. An experienced data quality supervisor will be assigned to perform daily quality examinations of a random sample of 10% of all the questionnaires administered during the day. Failed follow-up interviews will be re-attempted up to 3 times in the following 3 days at different hours with reasons being clearly recorded for each of the failures.

#### Data protection

All of the patients recruited in this study will be identified by a unique number. The patient number will be assigned to each participant once they have been recruited into this study. This patient number will be used from that point forward on all follow-up data. The hard copy documents of the study, including the informed consent of the patients and the project implementation diary, will be kept in locked files in the offices of the project assessment supervisor and the Principal Investigator. The electronic files, including the original EPI DATA files and Excel files, and interview recordings, will be backed-up on separate media and stored in a secure fling cabinet. Personal identifier will not be stored in the data set. Access to security passwords will be given only to the Principal Investigator and the Assessment Supervisor from the center of health management and technology, Anhui Medicine University. The center is independent from the sponsor and has no competing interests. Audits will be conducted by a dedicated team from the Health Sciences Division of Anhui Medical University to ensure data security conduct is adhered to.

#### Data analysis

The data collected will be used to compare the differences between the control and intervention groups as a whole and between subgroups in the intervention arm in terms of days of antibiotic use per episode of RTIs; times of re-visits to clinics and antibiotics re-prescription for the same RTI; quality of life; times of re-occurrence of RTIs and related health service seeking and antibiotics consumption for re-occurred infections; and scores of patients’ knowledge about and attitude toward antibiotics.

Estimation of statistical significance and confidence intervals will assume a type I error established in alpha=0.05, using the IBM SPSS V25 statistics package. Missing data will be treated as missing at random and imputed using a chained-equations multiple imputation model. Initial data analysis will consist of descriptive summaries intended to examine the patterns of the various measurements and check for normality of the continuous variables. And necessary transformations will be explored and selected, if necessary, to induce approximate normality. Regarding the numerical variables between two groups, *t*-test of independent samples for mean comparisons will be carried out. The chi-square test will be conducted for categorical variables (non-continuous). Lastly, no interim analysis is planned.

### Trial management

#### Trial steering committee

The trial is subject to the supervision of a trial steering committee (TSC) consisting of experts on patient education, primary health care provision and governance, and trial evaluation. This TSC will hold periodic (on a year base) and ad hoc (upon request by the trial implementation team) meetings to review trial progress and discuss solutions to outstanding issues.

#### Protocol amendments and deviations

Protocol amendments will first be sent to the trial sponsors for permission. Then, the application for ethical approval from the ECAMU will follow. Only after approval by ECAMU will the amended protocol be implemented and reported to the ISRCTN. All protocol violations and deviations will be reported and resolved according to funder and ethics board regulations.

#### Adverse event reporting

Given the nature of the intervention, we do not expect the occurrence of serious adverse events. Nonetheless, adverse events will be actively sought during each follow-up survey. All participants receive guidance to promptly inform the trial coordinator if they experience any events of concern. Additionally, the intervention is discontinued if a participant makes such a request, and if the investigator determines the participant’s inability to effectively utilize the intervention, or in the occurrence of a serious adverse event.

## Discussion

Both physicians and patients play important roles in antibiotic prescribing and uptake, while contemporary efforts in China have been focused primarily on doctors, with far less attention being put on patients. This study will determine the efficacy of a 12-message intervention to educate patients to reduce excessive antibiotic use. The intervention is innovative in a number of ways. First, the messages are designed to tackle existing problems identified through scoping literature reviews and field investigations [28–32]. Second, the messages are sent to patients at a unique time when they are suffering from RTIs and thus are at increased need for help in coping with their illness. Third, the messages are closely linked to the patients’ symptoms and diagnosis and are easily been felt as relevant and acceptable. Fourth, the intervention is facilitated by a computerized program and its implementation incurs little additional work load on the physicians. If proved effective, the intervention should be acceptable to both patients and physicians and scalable to other areas.

## Conclusion

This study will test the effectiveness of a 12-day message intervention for educating patients to reduce antibiotics use for symptomatic RTIs. This study will also provide information on message designing, modification, translation (of substitution variables), and sending facilitated by computer programs and ways in which it can be improved. If effective, the results will provide high-quality evidence to inform future translational research to scale up the intervention.

## Trial status

Protocol version 1.0(21/9/2022). The trial opened to recruitment on 1/12/2022 and recruitment is anticipated to be completed on 30/12/2026.

### Supplementary Information

**Additional file 1.** Questionnaire for patients: baseline.

**Additional file 2.** Questionnaire for patients: day 7.

**Additional file 3.** Questionnaire for patients: days 14 and 21.

**Additional file 4.** Questionnaire for patients: days 180 and 365.

**Additional file 5.** EQ-5D-5L.

**Additional file 6.** Patient consent record and contact details.

**Additional file 7.** SPIRIT Checklist for *Trials*.

## Data Availability

Data about the trial will be shared upon request to the corresponding author. Main results/findings from the trial will be disseminated via journal, workshops, and specific reports to relevant local and national policy makers.

## Abbreviations

RTIs: Respiratory tract infections
RCT: Randomized controlled trial
ABR: Antibiotic resistance
TMP: Tailored Message Package
UC: Usual care
ECAMU: Ethics Committee of Anhui Medical University
TSC: Trial Steering Committee
