# Supplementary material for: The effect of a tailored message package for reducing antibiotic use among respiratory tract infection patients in rural Anhui, China: a cluster randomized controlled trial protocol
Source: Trials. 2023 Oct 4;24:637. doi: 10.1186/s13063-023-07664-8 (PMC10548556; doi:10.1186/s13063-023-07664-8)
Supplement: Supplementary file 1 — Additional file 1. Questionnaire for patients: baseline. [file 13063_2023_7664_MOESM1_ESM.pdf]

Additional file A Questionnaire for interview of RTI patients: Baseline

A1: Eligibility criteria

A1a: Does the patient have a symptomatic RTI?

[ ] Yes (a1a=1)

[ ] No (a1a=0 and exclude from study)

A1b: Is the patient aged 18 years or older?

[ ] Yes (a1b=1)

[ ] No (a1b=0 and exclude from study)

A1c: Is today the first time the patient has sought treatment for this illness or a repeat visit?

[ ]First time consultation (a1c=1)

[ ]Repeated consultation (a1c=0 and exclude from study)

A1d: Can you read?

[ ]Yes(a1d=1)

[ ]No(a1d=0 and exclude from study)

Patient number:_______

A2: Participant Characteristic

A2a: Gender：

[ ]Female (a2a=1)

[ ]Male(a2a=2)

A2b: Date of Birth

([______]Year/[______]Month/[______]Day) or age[______]years

A2c: How many years of education have you completed?

[______]years [Write down the exact number]

A2d: Where do you live?

Name of town or village:[______________]

A2e:Have you lived outside your usual home residence for the past 12 months?

[ ] Yes (a2e=1)

[ ] No (a2e=0 or not applicable, and end of a2e)

A2e1: If a2e=1, how many months have you been away?

[______]months

A2e2:If a2e=1, Is the place where you have been living urban or rural?

[ ] Urban(a2e2=0)

[ ] Rural(a2e2=1)

[ ] Not applicable(a2e2=2)

A2f:What is your Household registration status for accessing health care benefits?

[ ] Urban(a2f=0)

[ ] Rural(a2f=1)

[ ] Other(a2f=2)

A2g: What type of insurance do you have, if any?

[ ]New rural cooperative Medical insurance scheme(a2g=0)

[ ]Urban Employee’s Medical Insurance(a2g=1)
[ ]Urban Residents’ Medical Insurance(a2g=2)
[ ]Not Having Medical Insurance(a2g=3)
[ ]Other Type of Insurance (a2g=4)

A3: Duration of illness and the severity
A3a: Duration of your current illness:
[_____] days
A3b:One a scale of 0 to 10,how sick do you feel?(where 10 is very sick and 0 is not sick)
[_____]

A4: patient’s symptoms
A4a:Pain ?
[ ] Yes (a4a=1)
[ ] No (a4a=0 and end of a4a)
A4a1: if a4a=1
[ ]Headache (a4a1=0)
[ ]Aching all-over (a4a1=1)
[ ]Chest pain (a4a1=2)
A4b: blocked/ runny nose?
[ ] Yes (a4b=1)
[ ] No (a4b=0 and end of a4b)
A4b1: if (a4b=1)
[ ]Blocked nose (a4b1=0)
[ ]Runny nose (clear/watery discharge) (a4b1=1)
[ ]Snotty nose (yellow/green discharge)(a4b1=2)
A4c: Coughing?
[ ] Yes (a4c=1)
[ ] No (a4c=0 and end of a4c)
A4c1: if a4c=1
[ ] Dry cough (a4c1=0)
[ ] Cough with white sputum (a4c1=1)
[ ] Cough with yellow/green sputum (a4c1=2)
A4d: Throat problems?
[ ] Yes (a4d=1)
[ ] No (a4d=0 and end of a4d)
A4d1: if a4d=1
[ ] Sore throat(a4d1=0)
[ ] Hyperemia/ swelling in throat(a4d1=1)
[ ] Hyperemia/ enlargement of tonsils(a4d1=2)
[ ] Pus on the tonsils(a4d1=4)
[ ] Swollen lymph glands (a4d1=4)
A4e: Breathing?
[ ] Yes (a4e=1)

[   ] No (a4e=0 and end of a4e)
A4e1: if a4e=1
[   ]Short of breath (a4e1=0)
[   ]Tight chest(a4e1=1)
[   ]Difficulty breathing (a4e1=3)
[   ]Must sit erect to breathe(a4e1=4)
[   ]Wet crackling in lungs(a4e1=5)
A4f:Ear symptoms?
[   ] Yes (a4f=1)
[   ] No (a4f=0 and end of a4f)
A4f1:if a4f=1
[   ]Blocked ears (a4f1=0)
[   ]Tinnitus (a4f1=1)
[   ]Pus/ fluid secretion (a4f1=2)
[   ]Earache (a4f1=3)
[   ]Loss of hearing (a4f1=4)
A4g:Fever?
[   ] Yes (a4g=1)
[   ] No (a4g=0 and end of a4g)
A4g1:If a4g=1,please fill in the degrees Celsius
[_____] ºC
A4g2:If you don't know the specific degrees Celsius, is low,moderate or high fever?
[   ]Low Fever (37.3-38 ºC) (a4g2=0)
[   ]Moderate Fever (38.1-39 ºC) (a4g2=1)
[   ]High Fever (>39 ºC) (a4g2=2)
A4i:Besides the above symptoms, do you have any other uncomfortable symptoms?
[   ] Yes (a4i=1)
[   ] No (a4i=0 and end of a4i)
A4i1:if a4i=1, what's wrong with you?
[_____] (please specify)

A5: Medicine
A5a: Have you had any treatment for your current illness before coming here?
[   ] Yes (a5a=1)
[   ] No (a5a=0 and end of a5a)
A5a1: If a5a=1,Where did you get these medicines?
[   ] Hospital or other clinic (a5a1=0)
[   ] Pharmacy (a5a1=1)
[   ] Family (a5a1=2)
[   ] Neighbors or friends (a5a1=3)
[   ] Home (a5a1=4)
[   ] Other(a5a1=5)[please specify][_____]
A5d: Have you used Antibiotics?
[   ] Yes (a5b=1)

[   ] No (a5b=0)

[   ]Do not know(b2a=2)

A5d1: :If a5d=1,How many days and times per day did you actually take a dose?

[   ]of days

[   ]times/day

A5c: How many times have you used antibiotics in the past 6 months?

[   ] 0 times(a5c=0)

[   ] 1-3 times(a5c=1)

[   ] 4-6 times(a5c=2)

[   ] > 6 times(a5c=3)A5d:Did your doctor prescribe medicine this time?

[   ] Yes (a5d=1)

[   ] No (a5d=0 and end of a5d)

A5e:if a5d=1,Did your doctor prescribe antibiotics this time?

[   ] Yes (a5e=1)

[   ] No (a5e=0 and end of a5e)

A5f:if a5e=1,what medicine was prescribed this time?

[_____]Medicine1,[_____]Single dose,[_____]frequency,[_____]Total dose

[_____]Medicine2,[_____]Single dose,[_____]frequency,[_____]Total dose

[_____]Medicine3,[_____]Single dose,[_____]frequency,[_____]Total dose

[_____]Medicine1,[_____]Single dose,[_____]frequency,[_____]Total dose

[_____]Medicine4,[_____]Single dose,[_____]frequency,[_____]Total dose

[_____]Medicine5,[_____]Single dose,[_____]frequency,[_____]Total dose

[_____]Medicine6,[_____]Single dose,[_____]frequency,[_____]Total dose

[_____]Medicine7,[_____]Single dose,[_____]frequency,[_____]Total dose

[_____]Medicine8,[_____]Single dose,[_____]frequency,[_____]Total dose

[_____]Medicine9,[_____]Single dose,[_____]frequency,[_____]Total dose
