# Supplementary material for: The effect of a tailored message package for reducing antibiotic use among respiratory tract infection patients in rural Anhui, China: a cluster randomized controlled trial protocol
Source: Trials. 2023 Oct 4;24:637. doi: 10.1186/s13063-023-07664-8 (PMC10548556; doi:10.1186/s13063-023-07664-8)
Supplement: Supplementary file 2 — Additional file 2. Questionnaire for patients: day 7. [file 13063_2023_7664_MOESM2_ESM.pdf]

Additional file B Questionnaire for telephone interview of RTI patients: Day 7

Patent number:______

B1:Duration of illness and the severity

B1a: How is your illness you sought help in [name of the health centre] 7 days ago?

[ ]Recovered completely (b1a=0)

[ ]Better (b1a=1)

[ ]Almost the same (b1a=2)

[ ]Worse(b1a=3)

B1b: If b1a=0, which day did you recovered completely after this illness?

[ ]days

B1c:One a scale of 0 to 10,how sick do you feel?(where 10 is very sick and 0 is not sick)

[_____]

B2: Medicine Consumption

B2a:Have you prescribed antibiotics from the health center in your initial consultation ?

[ ]No(b2a=0)

[ ]Yes(b2a=1)

[ ]Do not know(b2a=2)

B2a1:If b2a=1,How many days and times per day did you actually take a dose?

[ ]of days

[ ]times/day

B2a2:What was the dose did you take every time?

[ ]

B2a3:Have you stooped taking the antibiotic that were prescribed?

[ ]No(b2a3=0)

[ ]Yes(b2a3=1)

B3: attitude and understanding of intervention message

B3a: Have you received any messages about your illness from the health center during the past 7 days ?

[ ] Yes (b3a=1)

[ ] No (b3a=0 and end of b3)

B3b:If b3a=1,have you read the messages?

[ ] Yes (b3b=1)

[ ] No (b3b=0 and end of b3)

B3c:If b3b=1,Are the messages easy to understand?

[ ] Easy (b3c=1)

[ ] Not easy(b3c=0 and end of b3c)

B3d: Are the messages useful?

[ ] Yes (b3d=2)

[ ] Unclear(b3d=1)

[   ] No (b3d=0 and end of b3d)

B3e: Have you practiced anything as the messages had suggested?

[   ] Yes (b3e=1)

[   ] No (b3e=0 and end of b3e)
