# Supplementary material for: The effect of a tailored message package for reducing antibiotic use among respiratory tract infection patients in rural Anhui, China: a cluster randomized controlled trial protocol
Source: Trials. 2023 Oct 4;24:637. doi: 10.1186/s13063-023-07664-8 (PMC10548556; doi:10.1186/s13063-023-07664-8)
Supplement: Supplementary file 3 — Additional file 3. Questionnaire for patients: days 14 and 21. [file 13063_2023_7664_MOESM3_ESM.pdf]

Additional file C Questionnaire for telephone interview of RTI patients: Day 14 & 21

Patient number:_______

C1: Duration of illness and the severity

C1a: How is your illness you sought help in [name of the health centre] 14/21 days ago?

[ ]Recovered completely (c1a=0)

[ ]Better (c1a=1)

[ ]Almost the same (b1a=2)

[ ]Worse(c1a=3)

C1b:If c1a=1, which days did you recovered completely after this illness?

[ ]days

C1c:One a scale of 0 to 10,how sick do you feel?(where 10 is very sick and 0 is not sick)

[_____]

C2: Antibiotics Consumption

C2a:Have you prescribed antibiotic from the health center in your initial consultation (Only ask patient telephone questionnaire–Day 14)?

[ ]No(c2a=0)

[ ]Yes(c2a=1)

[ ]Do not know(c2a=2)

C2a1:If c2a=1,How many days and times per day did you actually take a dose?

[ ]of days

[ ]times/day

C2a2:What was the dose did you take every time?

[ ]

C2a3:Have you stooped taking the antibiotics/anti-inflammation medicine that were prescribed?

[ ]No(c2a3=0)

[ ]Yes(c2a3=1)

C2b:Have you ever used drips since your first visit to the health center(Only ask patient telephone questionnaire–Day 21)?

[ ] Yes (c2b=1)

[ ] No (cc2b=0 and end of c2b)

C2b1:(if c2b=1) How many days in which you used drips?

[______]days

C3: Times of re-visits and antibiotic re-prescription

C3a:Have you been to [name of the health center] again since the first visit for your current illness?

[ ]No(c3a=0 and end of c3)

[ ]Yes(c3a=1)

C3a1:If c3a =1,How many times have you been to [name of the health center] again since the first visit for your current illness?

[ ]times

C3a2: Have you prescribed any antibiotics for the first time(Note: exclude the initial consultation)?

[ ]No(c3a2=0)

[ ]Yes(c3a2=1)

[ ]Do not know(c3a2=2)

C3a21:If c3a2=1,How many days and times per day did you actually take a dose?

[ ]of days

[ ]times/day

C3a3: Have you prescribed any antibiotics for the second time(Note: exclude the initial consultation)?

[ ]No(c3a3=0)

[ ]Yes(c3a3=1)

[ ]Do not know(c3a2=2)

C3a21:If c3a3=1,How many days and times per day did you actually take a dose?

[ ]of days

[ ]times/day

C3a4: Have you prescribed any antibiotics for the third time(Note: exclude the initial consultation)?

[ ]No(c3a4=0)

[ ]Yes(c3a4=1)

[ ]Do not know(c3a44=2)

C3a41:If c3a4=1,How many days and times per day did you actually take a dose?

[ ]of days

[ ]times/day

C4:Additional Health Care Utilisation (Only ask patient telephone questionnaire–Day 21)

C4a: Have you ever bought medicine from the drugstore since the first visit heath center for your current illness?

[ ] Yes (c4a=1)

[ ] No (c4a=0 and end of c4a)

C4a1:if c4a=1,how many days after your illness did you go to the drugstore for the first time?

[______]days

C4a2:How many times have you been there?

[______]times

C4a3: Have you bought any antibacterial drugs?

[ ] Yes (c4a3=1)

[ ] No (c4a3=0)

C4b: Have you seen a doctor elsewhere for the same disease since you went back from the health center last time?

[ ] Yes (c4b=1)

[ ] No (c4b=0 and end of c4b)

C4b1:[______]Clinic / hospital1

C4b11: How many days did you go for the first time after you got sick?

[______]days

C4b12: How many times have you been there?

[______]times

C4b13: Did you get the medicine?

[ ] Yes c4b13=1)

[ ] No (c4b13=0)

C4b14: Did you get drip?

[ ] Yes (c4b14=1)

[ ] No (c4b14=0)

C4b15: Have you bought any antibacterial drugs?

[ ] Yes (c4b15=1)

[ ] No (c4b15=0)

C4b2:[______]Clinic / hospital2

C4b21: How many days did you go for the first time after you got sick?

[______]days

C4b22: How many times have you been there?

[______]times

C4b23: Did you get the medicine?

[ ] Yes (c4b23=1)

[ ] No (c4b23=0)

C4b24: Did you get drip?

[ ] Yes (c4b24=1)

[ ] No (c4b24=0)

C4b25: Have you bought anyantibacterial drugs?

[ ] Yes (c4b25=1)

[ ] No (c4b25=0)

C5: Attitude and understanding of intervention message (Only ask patient telephone questionnaire–Day 14)

C5a:Have you received any messages about your illness from the health center during the past 14 days ?

[ ] Yes (c5a=1)

[ ] No (c5a=0 and end of c5)

C5b:If c5a=1,have you read the messages?

[ ] Yes (c5b=1)

[ ] No (c5b=0 and end of c5)

C5c:If c5b=1,Are the messages easy to understand?

[ ] Easy (c5c=1)

[   ] Not easy(c5c=0 and end of c5c)

C5d: Are the messages useful?

[   ] Yes (c5d=2)

[   ] Unclear(c5d=1)

[   ] No (c5d=0 and end of c5d)

C5e: Have you practiced anything as the messages had suggest?

[   ] Yes (c5e=1)

[   ] No (c5e=0 and end of c5e)
