# Supplementary material for: The effect of a tailored message package for reducing antibiotic use among respiratory tract infection patients in rural Anhui, China: a cluster randomized controlled trial protocol
Source: Trials. 2023 Oct 4;24:637. doi: 10.1186/s13063-023-07664-8 (PMC10548556; doi:10.1186/s13063-023-07664-8)
Supplement: Supplementary file 4 — Additional file 4. Questionnaire for patients: days 180 and 365. [file 13063_2023_7664_MOESM4_ESM.pdf]

**Additional file D Questionnaire for telephone interview of RTI patients: Day 180 & 365**

Patent number:______

**D1 Knowledge about and attitude toward antibiotic**

D1a:Do you always wash your hands when you go home from outside?

[ ]No(d1a=0)

[ ]Yes(d1a=1)

D1b: If someone around you infected RTI, would you try to avoid them?

[ ]No(d1b=0)

[ ]Yes(d1b=1)

D1c:Do you think the following measures can prevent respiratory diseases such as colds, sinusitis, pharyngitis, tracheitis, etc? (Read the options in order,1=Yes, 0= No)

[ ]No smoking
[ ] Avoid contact with smoke and dust
[ ] Eat more fruits, vegetables, and grains
[ ]Avoid going to crowded places
[ ] Do more physical exercise and physical activity
[ ] Avoid contact with patients
[ ] Influenza vaccination
[ ] Avoid excessive fatigue
[ ] Maintain mental pleasure
[ ] Regular window opening for ventilation
[ ] Cover the mouth and nose when coughing or sneezing
[ ] Other (please specify)_____________

D1d: When you see a doctor at ordinary times, do you take the initiative to ask the doctor to prescribe antibiotics ?

[ ]No(d1d=0)

[ ]Yes(d1d=1)

[ ]No longer remember(d1d=2)

D1e: When do you usually stop taking the antibiotics prescribed by the doctor? (1=Yes, 0= No)

[ ]Stop eat when symptoms improve(d1e =0)

[ ]Stop eat when the symptoms are clear(d1e =1)

[ ]Drug withdrawal due to poor effect(d1e =2)

[ ]Adverse reaction withdrawal(d1e =3)

[ ]Keep eating(d1e =4)

[ ]Other(d1e =5) (please specify)______________

D1f:What would you do if the doctor told you to leave without any medicine?

[ ]Unhappy(d1f=0)

[ ]See another doctor(d1f =1)

[ ]Do as the doctor says(d1f =2)

[ ]Other(d1f =3) (please specify)__________

D1j: Do you often reserve some antibacterial drugs in your home?

[ ]No(d1j=0)

[ ]Yes(d1j=1)D1h :Which infections do you think antibiotics / anti-inflammatory drugs are useful for? (1=Yes, 0= No)

[ ]Bacterial infection

[ ]Viral infection

[ ] Do not know

D1i: Can an antibiotic kill or control a virus?

[ ] No (d1i=0)

[ ] Yes (d1i=1)

[ ] Do not know(d1i=2)

D1j: What do you think are the disadvantages of using antibiotics? (1=Yes, 0= No)

[ ]Drug resistance

[ ]Economic burden

[ ]Allergic reactions

[ ]Liver / kidney damage

[ ]Hearing impairment

[ ]Anemia and other blood system problems

[ ]Dizziness / headache

[ ]Gastrointestinal discomfort

[ ]Don't know

[ ]Others (please specify)______________

D1k: Will frequent use of antibioticss reduce the efficacy?

[ ] No(d1k=0)

[ ] Yes (d1k=1)

[ ] Do not know(d1k=2)

D1l: Can you accept not seeing a doctor immediately when you are infected with respiratory diseases?

[ ] No (d11l=0)

[ ] Yes (d1l1=1)

D1m:How many days do you think you will go to see a doctor when you are infected with respiratory diseases? (1=Yes, 0= No)

[ ] First day [ ] Second day

[ ] Third day

[ ] Fourth day

[ ] >fourth day

**D2 Health service seeking and antibiotics consumption for re-occurred infections**

D2a:Did you have re-occurrence of RTIs in the past six months?

[ ] No (d2a=0)

[ ] Yes (d2a=1 and skip d2a)

D2a1:Times of re-occurrence of RTIs in the past six months

[______] times

D2b: First time of re-occurrence of RTIs

D2b1:Have you saw a doctor?

[ ] Yes (d2b1=1)

[ ] No (d2b1=0 and end of d2b1)

D2b2: How many days after your illness did you go to the doctor?

[______] days

D2b3: Did you used antibiotics?

[ ] Yes (d2b3=1)

[ ] No (d2b3=0)

D2b31:If d2b3=1,How many days of eating antibiotics?

[______]days

D2b4: Have you ever get drip?

[ ] Yes (d2b4=1)

[ ] No (d2b4=0)

D2b41:If b1b4=1,How many days in which you used drips?

[______]days

D2c: Second time of re-occurrence of RTIs

D2c1:Have you saw a doctor?

[ ] Yes (d2c1=1)

[ ] No (d2c1=0 and end of d2c1)

D2c2: How many days after your illness did you go to the doctor?

[______]days

D2c3: Did you used antibiotics?

[ ] Yes (d2c3=1)

[ ] No (d2c3=0)

D2c31:If d2c3=1,how many days of eating antibiotics?

[______]days

D2c4: Have you ever get drip?

[ ] Yes (d2c4=1)

[ ] No (d2c4=0)

D2c41:If d2c4=1,How many days in which you used drips?

[______]days

D3c: third time of re-occurrence of RTIs

D3c1:Have you saw a doctor?

[ ] Yes (d3c1=1)

[ ] No (d3c1=0 and end of d3c1)

D3c2: How many days after your illness did you go to the doctor?

[______]days

D3c3: Did you used antibiotics?

[ ] Yes (d3c3=1)

[ ] No (d3c3=0)

D3c31:If d3c3=1,how many days of eating antibiotics?

[______]days

D3c4: Have you ever get drip?

[ ] Yes (d3c4=1)

[ ] No (d3c4=0)

D3c41:If d3c4=1,How many days in which you used drips?

[_______]days
