# Supplementary material for: The effect of a tailored message package for reducing antibiotic use among respiratory tract infection patients in rural Anhui, China: a cluster randomized controlled trial protocol
Source: Trials. 2023 Oct 4;24:637. doi: 10.1186/s13063-023-07664-8 (PMC10548556; doi:10.1186/s13063-023-07664-8)

Additional file E EQ-5D -5L

Health Questionnaire

English version for the UK

VERSION FOR INTERVIEWER ADMINISTRATION

INTRODUCTION

(Note to interviewer: please read the following to the respondent.)

We are trying to find out what you think about your health. I will explain what to do as I go along, but please interrupt me if you do not understand something or if things are not clear to you. There are no right or wrong answers. We are interested only in your personal view.

First, I am going to read out some questions. Each question has a choice of five answers. Please tell me which answer best describes your health TODAY.

Do not choose more than one answer in each group of questions.

(Note to interviewer: first read all five options for each question. Then ask the respondent to choose which one applies to him/herself. Repeat the question and options if necessary. Mark the appropriate box under each heading. You may need to remind the respondent regularly that the timeframe is TODAY.)

EQ-5D DESCRIPTIVE SYSTEM

MOBILITY

E1a: I would like to ask you about mobility. Would you say that:

[ ] You have <u>no</u> problems in walking about? (e1a=0)

[ ] You have <u>slight</u> problems in walking about? (e1a=1)

[ ]You have <u>moderate</u> problems in walking about? (e1a=2)

[ ]You have <u>severe</u> problems in walking about? (e1a=3)

[ ]You are <u>unable to</u> walk about? (e1a=4)

SELF-CARE

E1b: I would like to ask you about self-care. Would you say that:

[ ] You have <u>no</u> problems washing or dressing yourself?(e1b=0)

[ ] You have <u>slight</u> problems washing or dressing yourself?(e1b=1)

[ ] You have <u>moderate</u> problems washing or dressing yourself?(e1b=2)

[ ] You have <u>severe</u> problems washing or dressing yourself?(e1b=3)

[ ] You are <u>unable to</u> wash or dress yourself?(e1b=4)

USUAL ACTIVITIES

E1c: I would like to ask you about usual activities, for example work, study, housework, family or leisure activities. Would you say that:

[ ]You have <u>no</u> problems doing your usual activities?(e1c=0)

[ ]You have <u>slight</u> problems doing your usual activities?(e1c=1)

[ ]You have <u>moderate</u> problems doing your usual activities?(e1c=2)

[ ]You have <u>severe</u> problems doing your usual activities?(e1c=3)

[ ]You are <u>unable to</u> do your usual activities?(e1c=4)

PAIN / DISCOMFORT

E1d: I would like to ask you about pain or discomfort. Would you say that:

[ ]You have <u>no</u> pain or discomfort? (e1d=0)

[ ]You have <u>slight</u> pain or discomfort?(e1d=1)

[ ]You have <u>moderate</u> pain or discomfort?(e1d=2)

[ ]You have <u>severe</u> pain or discomfort? (e1d=3)

[ ]You have <u>extreme</u> pain or discomfort?(e1d=4)

ANXIETY / DEPRESSION

E1e: I would like to ask you about anxiety or depression. Would you say that:

[ ]You are <u>not</u> anxious or depressed?(e1e=0)

[ ]You are <u>slightly</u> anxious or depressed?(e1e=1)

[ ]You are <u>moderately</u> anxious or depressed?(e1e=2)

[ ]You are <u>severely</u> anxious or depressed?(e1e=3)

[ ]You are <u>extremely</u> anxious or depressed? (e1e=4)

EQ-5D VAS

- **Now, I would like to ask you to say how good or bad your health is TODAY.**

- **I would like you to try to picture in your mind a scale that looks like a thermometer.**
  (Note to interviewer: if interviewing face-to-face, please show the person the VAS scale.)

- **The best health you can imagine is marked 100 (one hundred) at the top of the scale and the worst health you can imagine is marked 0 (zero) at the bottom.**

- **I would now like you to tell me the point on this scale where you would put your health TODAY.**
  (Note to interviewer: mark the scale at the point indicating the respondent's 'health today'. Now, please write the number you marked on the scale in the box below.)

THE RESPONDENT'S HEALTH TODAY = [ ]

Thank you for taking the time to answer these questions.

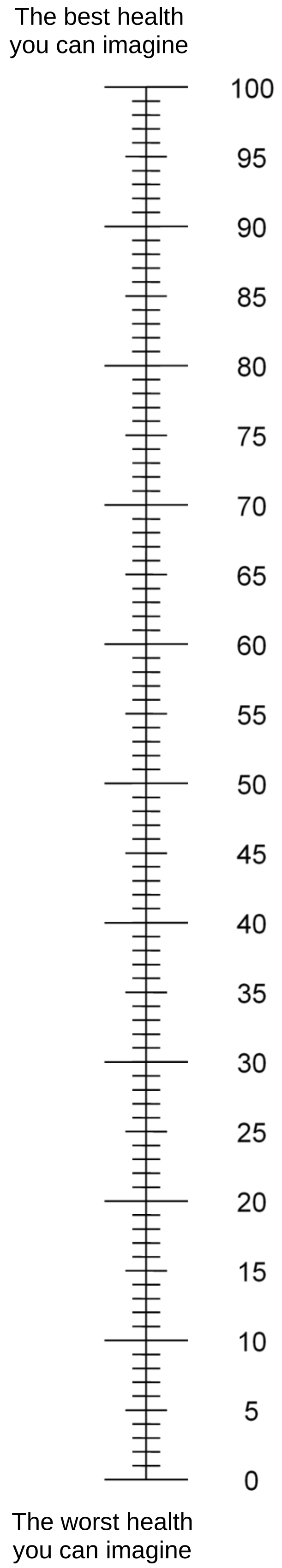

Supplement: Supplementary file 5 — Additional file 5. EQ-5D-5L. [file 13063_2023_7664_MOESM5_ESM.pdf]
