# Supplementary material for: The effect of a tailored message package for reducing antibiotic use among respiratory tract infection patients in rural Anhui, China: a cluster randomized controlled trial protocol
Source: Trials. 2023 Oct 4;24:637. doi: 10.1186/s13063-023-07664-8 (PMC10548556; doi:10.1186/s13063-023-07664-8)
Supplement: Supplementary file 6 — Additional file 6. Patient consent record and contact details. [file 13063_2023_7664_MOESM6_ESM.pdf]

Additional file F Patient Consent Record & Contact Details

Dear patient

We invite you to participate in the project named "Cluster randomized controlled trial to assess a tailored intervention to reduce antibiotic using in primary care" funded by the National Natural Science Foundation of China and Anhui provincial Universities Scientific Research Project. This study has been approved by the Ethics Committee of Anhui Medical University. In order to protect your rights, please read the following statement of consent.

Consent statement (and see overleaf)

1.	I understand what this study is about and how I am being asked to participate in it.	
2.	I understand that taking part in this research is voluntary and that I am free to leave the study at any time, without giving any reason, and without my medical care or rights or those of my family being affected.	
3.	I understand that any information I provide will be anonymised by removing all identifying details and will be kept strictly confidential and used only for research purposes. After the study, the anonymised information will be made publicly available for potential further research, but it will not be possible to identify me from the data.	
4.	My consent is based on the Anhui Medical University complying with their duties and obligations under the Data Protection Act.	

I understand the above statements and am fully aware of the risks and benefits that may be incurred in this study. I voluntarily participate in this study.

Patient number: ___________________________
Participant Contact Details

Address: ______________________________
Phone number1: _______________________
Phone number2: _______________________

Participant Consent (when the participant is not able to sign for themselves, the researcher will sign on their behalf to record their consent):

____________________	____________	____________________
Name of Participant	Date	Signature
____________________	____________	____________________
Name of Family member	Date	Signature
____________________	____________	____________________
Name of Person taking consent	Date	Signature

If the patient refuses the above informed consent statement, ask why he/she chooses not to participate.

Reason for refusal: ____
